# Incidence and Associated Risk Factors in the Development of Carfilzomib-Induced Cardiovascular Toxicity

**DOI:** 10.3390/curroncol33080471

**Published:** 2026-08-08

**Authors:** Noor Lad, Jayda Esplund, Dennis Grauer, Shebli Atrash, Prerna Mewawalla, Tejaswi Gadela, Charles Porter, Muhammad Mushtaq, Jeries Kort, Donald C. Moore, Al-Ola Abdallah, Zahra Mahmoudjafari, Jordan Snyder

**Affiliations:** 1 Division of Hematologic Malignancies & Cellular Therapeutics, University of Kansas Medical Center, Westwood, KS 66205, USA; 2 Department of Pharmacy Practice, The University of Kansas School of Pharmacy, Kansas City, KS 66047, USA; 3 Levine Cancer Center, Atrium Health, Wake Forest University School of Medicine, Charlotte, NC 28262, USA; 4 Division of Hematology and Cellular Therapy, AHN Cancer Institute, Allegheny Health Network, Pittsburgh, PA 15212, USA; 5 Department of Cardiology, University of Kansas Medical Center, Kansas City, KS 66160, USA; cbporter@kumc.edu

**Keywords:** multiple myeloma, carfilzomib, cardiovascular adverse events, cardiotoxicity, cardio-oncology, risk factors

## Abstract

Background: Carfilzomib, a second-generation proteasome inhibitor, is widely used in multiple myeloma (MM) treatment but has been associated with cardiovascular adverse events (CVAEs). Real-world data evaluating the incidence and risk factors are limited. Methods: We conducted a multicenter retrospective cohort study of 385 adult MM patients treated with carfilzomib between January 2020 and August 2024 at three U.S. institutions. The primary objective of this study was to determine the incidence of carfilzomib-associated CVAEs. The secondary objectives included the identification of risk factors, characterization of cardiovascular events, and time-to-onset analysis. Results: Carfilzomib-associated CVAEs occurred in 25 patients (6.5%). The median time to event was 114 days. Heart failure was the most common manifestation (86%), followed by arrhythmias (32%) and acute coronary syndromes (8%). Patients with baseline heart failure had a significantly increased risk of CVAEs (HR 1.61, *p* = 0.042), whereas arrhythmias showed a trend toward significance. Traditional cardiovascular risk factors were not independently associated with an increased risk. CVAEs were associated with numerically inferior overall survival (45.7 vs. 97.3 months; HR 1.316, 95% CI 0.728–2.377, *p* = 0.361). Partial recovery of the left ventricular ejection fraction was observed following treatment discontinuation. Conclusion: Carfilzomib-associated CVAEs were infrequent but clinically meaningful in this multicenter cohort. These findings support consideration of a risk-adapted cardio-oncology approach, particularly in patients with pre-existing cardiac dysfunction, where closer surveillance and early intervention may help mitigate clinically significant cardiotoxicity.

## Simple Summary

Carfilzomib is an effective treatment for multiple myeloma but can sometimes cause serious heart and blood vessel problems. We reviewed the medical records of 385 adults treated with carfilzomib at three United States cancer centers to better understand how often these complications occur and which patients are at the greatest risk. Heart-related complications developed in 6.5% of patients, with heart failure being the most common event. Patients with a history of heart failure before starting treatment were at the highest risk of developing cardiovascular complications. These events occurred at varying times during treatment, highlighting the need for ongoing monitoring rather than only early assessment. Our findings support careful cardiovascular evaluation before starting carfilzomib and continued monitoring throughout treatment, particularly for patients with pre-existing heart disease, to improve the safe use of this therapy.

## 1. Background

Multiple myeloma (MM) accounts for 1% of all cancers and approximately 10% of all hematologic malignancies, making it the second most common hematologic malignancy [1,2,3]. The median age of patients at the time of diagnosis is 66 years [4,5]. The overall management of MM has evolved with the introduction of new and novel agents, including proteasome inhibitors (PI), immunomodulatory drugs, and monoclonal antibodies. Although these advances have significantly improved survival, they have also introduced clinically meaningful and sometimes treatment-limiting toxicities, particularly in the form of cardiovascular complications.

The backbone of MM therapy often includes PIs, such as carfilzomib and bortezomib. These drugs block the proteasome, a complex that degrades unneeded or damaged proteins within cells [6,7]. By inhibiting this process, PIs cause an accumulation of proteins, leading to cell stress and ultimately cell death [6,7]. MM cells produce excessive amounts of abnormal proteins, making them particularly sensitive to proteasome inhibition [7,8]. This selective toxicity helps to effectively target and kill myeloma cells while sparing most normal cells.

Carfilzomib, a second-generation irreversible PI approved in 2013, has become widely integrated into frontline and relapsed MM regimens, including in transplant-eligible and -ineligible populations [9]. However, randomized trials, including ASPIRE and ENDEAVOR, have consistently demonstrated an increased incidence of cardiovascular adverse events (CVAEs), raising concerns regarding its safety.

In the phase III ASPIRE trial, carfilzomib combined with lenalidomide and dexamethasone (KRd) significantly improved median progression-free survival (PFS) compared with Rd (26.3 vs. 17.6 months) but was associated with increased rates of grade ≥3 hypertension, heart failure, and ischemic events (6.4%, 4.3%, and 3.8% respectively) [10]. Similarly, in ENDEAVOR, carfilzomib plus dexamethasone (Kd) improved PFS (18.7 vs. 9.4 months) and overall survival (47.6 vs. 40 months) compared with bortezomib-based therapy, at the expense of higher cardiovascular toxicity [11,12]. Collectively, these findings establish a clear efficacy–toxicity trade-off, with cardiovascular complications emerging as a clinically significant limitation of carfilzomib therapy.

Given that MM predominantly affects older patients, many with pre-existing cardiovascular comorbidities, the external validity of clinical trial safety data remains uncertain [13,14]. Despite these concerns, real-world data characterizing CVAEs in patients with baseline cardiovascular risk remain limited, particularly outside the controlled environment of clinical trials. Given the aging population affected by MM, understanding the interplay between carfilzomib treatment and preexisting cardiac conditions is crucial. Accordingly, we conducted a retrospective analysis to quantify the incidence of CVAEs in patients treated with carfilzomib and to determine whether pre-existing cardiovascular risk factors independently predicted cardiotoxicity in routine clinical practice.

## 2. Methods

This was a multicenter, retrospective cohort study of adult patients with MM treated with carfilzomib between 1 January 2020 and 1 August 2024, at the University of Kansas Cancer Center, Allegheny Health Network Cancer Institute, and Levine Cancer Institute/Wake Forest University. Data were extracted from electronic medical records (EPIC^®^) and managed using a secure REDCap™ database. The primary objective of this study was to determine the incidence of carfilzomib-associated CVAEs. Secondary objectives included identification of risk factors for CVAEs, characterizing the spectrum of cardiovascular events, and evaluating the time to onset.

Because this was a retrospective observational study, no standardized study-specific cardiovascular eligibility criteria (e.g., minimum left ventricular ejection fraction threshold) were applied for initiation of carfilzomib therapy. Treatment decisions, including the use of carfilzomib, were made at the discretion of the treating hematologist/oncologist according to institutional practice and individual patient characteristics.

Carfilzomib-associated CVAEs were defined as the development of a new or worsening cardiovascular adverse event after initiation of carfilzomib, with a temporal association and no alternative clearly identifiable cause, as determined by clinical documentation. Cardiovascular events were identified through review of physician documentation, diagnostic testing, and hospitalization records.

CVAEs included new-onset or worsening heart failure characterized by clinical signs and symptoms (e.g., dyspnea, orthopnea, volume overload) in association with objective evidence of cardiac dysfunction, including left ventricular systolic dysfunction on echocardiography and/or hemodynamic congestion consistent with cardiogenic pulmonary edema, as well as a decrease in left ventricular ejection fraction when imaging data were available. Additional CVAEs included clinically significant arrhythmias requiring medical intervention, ischemic events (including myocardial infarction or unstable angina), hypertensive emergencies, and other clinically significant cardiovascular events. Baseline cardiovascular disease was defined according to documented medical history before initiation of carfilzomib. Because disease severity and treatment status were not uniformly documented, these variables could not be analyzed separately. In patients with pre-existing cardiovascular disease, CVAEs were defined as documented clinical worsening temporally associated with carfilzomib exposure. Although CVAEs were defined based on clinician documentation and objective findings, standardized criteria such as CTCAE grading and ESC-defined cancer therapy-related cardiac dysfunction were not uniformly applied, which may introduce variability in event ascertainment.

Statistical analyses were performed using SPSS software (version 28). Categorical variables were compared between patients with and without CVAEs using the chi-square test or Fisher’s exact test. Continuous variables that were not normally distributed were analyzed using the Mann–Whitney U test. Descriptive statistics were used to summarize baseline characteristics, prior therapies, and cardiovascular outcomes.

Risk factor identification for carfilzomib-associated CVAEs was performed using Cox proportional hazards regression to account for the time-to-event from carfilzomib initiation to first CVAE. Multivariable Cox proportional hazards models were used to identify independent predictors of CVAEs. Candidate baseline covariates were selected a priori based on the published cardio-oncology literature, established risk factors for cancer therapy-related cardiovascular toxicity, and their clinical relevance to patients receiving proteasome inhibitor therapy. Variables considered for analysis included age, baseline cardiovascular disease (including heart failure and arrhythmias), hypertension, hyperlipidemia, prior therapies, and treatment regimen. Variable selection was based on biological plausibility and previously reported associations with carfilzomib-associated cardiovascular toxicity rather than statistical significance in univariate analyses.

Overall survival was evaluated using Kaplan–Meier methods and compared using the log-rank test. Hazard ratios (HRs) with 95% confidence intervals (CIs) were estimated using Cox proportional hazards regression. A two-sided *p*-value < 0.05 was considered statistically significant.

The study protocol was approved by the Institutional Review Board of the University of Kansas Health System (IRB 00160482; approved 10 August 2024).

## 3. Results

A total of 385 patients were included in the final analysis, of whom 25 (6.5%) developed CVAEs. The median age at the time of carfilzomib initiation was 66 (31–88) years. Of the cohort, 206 patients (57%) were male, and 282 (77%) identified as White. The most common baseline cardiovascular risk factors were hypertension (56%), dyslipidemia (42%), tobacco use (39%), and chronic renal failure (29%). Baseline heart failure and arrhythmia were present in 47 (12%) and 65 (17%) patients, respectively. The median baseline left ventricular ejection fraction (LVEF) was 60% (40–76%) (Table 1).

All patients had received ≥1 prior line of therapy before carfilzomib initiation. Most patients were treated with immunomodulatory agents (90%) and proteasome inhibitors (89%). Other common prior therapies included alkylating agents (61%) and lymphodepleting chemotherapy (e.g., fludarabine/cyclophosphamide, typically in the context of cellular therapy) (6%) (Table 2).

The cumulative incidence of carfilzomib-associated CVAEs was 6.5%. The median time to onset of a cardiovascular adverse event (CVAE) was 157 days (3–1323 days), suggesting both early and delayed presentations. In patients who experienced a CVAE, heart failure was the predominant event (86%), followed by arrhythmias (32%), pulmonary edema (20%), and acute coronary syndromes (8%). Heart failure events were characterized by clinical signs and symptoms of volume overload and/or dyspnea in association with objective evidence of cardiac dysfunction, including echocardiographic abnormalities and/or imaging evidence of pulmonary congestion. At the time of CVAE, patents demonstrated a median absolute decline in LVEF from baseline of 35%. Following discontinuation of carfilzomib, a subset of patients demonstrated partial recovery of LVEF from the nadir, with a median improvement of 20%, suggesting that carfilzomib-associated cardiac dysfunction was at least partially reversible in many patients, although the degree of recovery was variable (Table 3).

Cardiac biomarkers were frequently elevated at the time of cardiovascular adverse events. Among patients with available data, the median NT-proBNP was 9329 pg/mL (range, 412–17,500; *n* = 5), and the median BNP was 1259 pg/mL (range, 88–5771; *n* = 16). These findings are based on a limited subset of patients and should be considered exploratory, as biomarker assessment was not uniformly performed, and no comparator group was available.

In the primary time-to-event analysis, multivariable Cox proportional hazards regression was used to identify factors associated with the development of carfilzomib-associated CVAEs. Baseline heart failure was independently associated with an increased risk of CVAEs (HR 1.61, 95% CI 1.02–2.56; *p* = 0.042)**.** Baseline arrhythmias demonstrated a trend toward increased risk but did not reach statistical significance (HR 1.47, 95% CI 0.99–2.20; *p* = 0.055). Hypertension and hyperlipidemia were not associated with an increased risk (HR 1.07; *p* = 0.694 and HR 0.84; *p* = 0.328, respectively) (Table 4).

**Figure 1:**
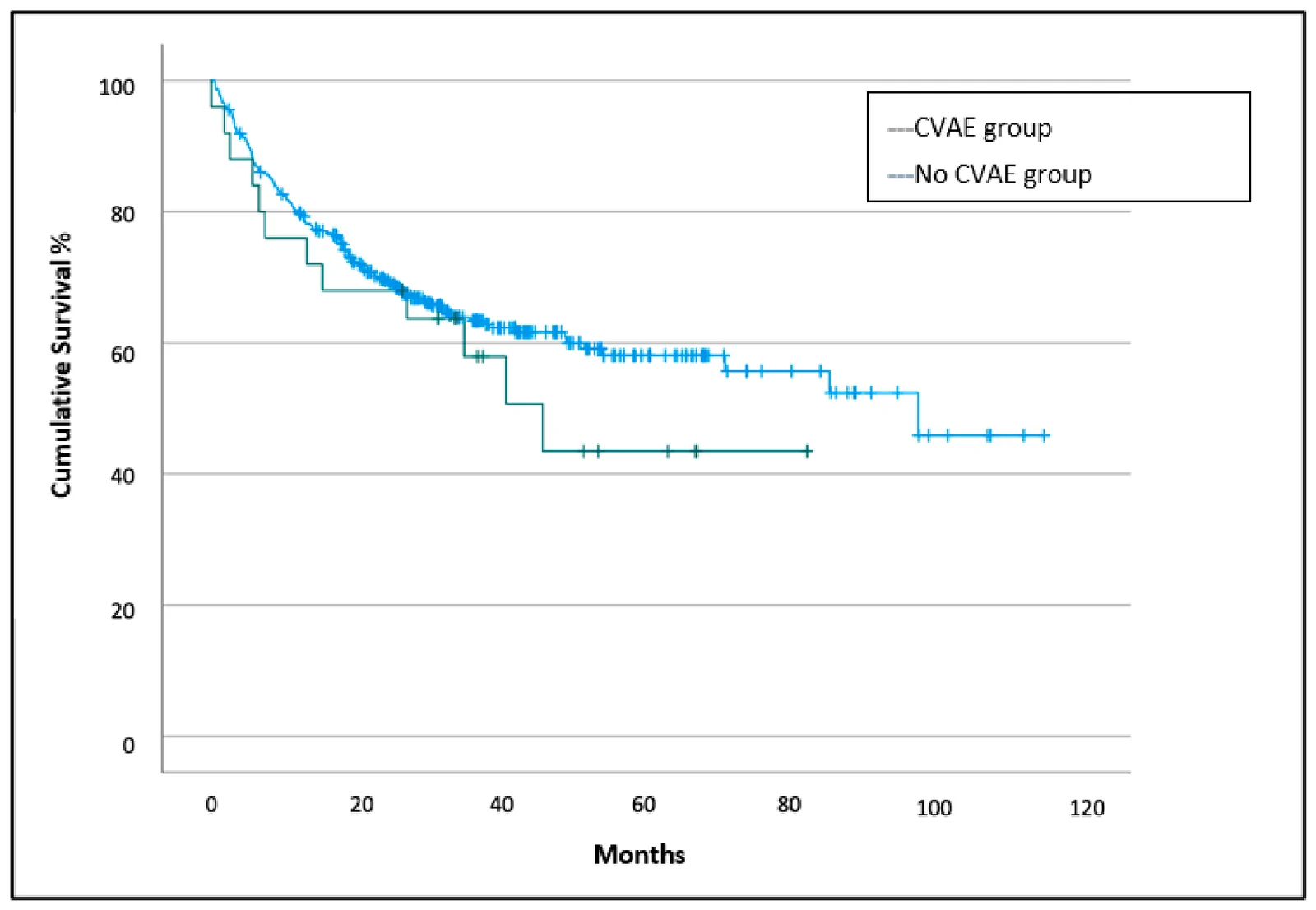
Kaplan–Meier overall survival curves in patients receiving carfilzomib stratified by development of CVAEs. Patients who developed CVAEs demonstrated numerically shorter overall survival compared with those without cardiotoxicity (HR 1.316, 95% CI 0.728–2.377; log-rank *p* = 0.361).

Patients who developed CVAEs had a numerically shorter median overall survival (45.7 vs. 97.3 months) (HR 1.316, 95% CI 0.728–2.377, *p* = 0.361) (Figure 1). During follow-up, 12 patients (48%) in the cardiotoxicity cohort died, predominantly due to progressive multiple myeloma (75%), with the remaining deaths attributed to other causes (25%). Notably, no deaths were directly attributable to carfilzomib-associated cardiotoxicity.

These findings underscore that CVAEs, while relatively infrequent, are strongly enriched in patients with pre-existing cardiac disease and are associated with numerically inferior survival outcomes.

## 4. Discussion

In this multicenter, retrospective study, we found that clinically significant carfilzomib-associated CVAEs occurred in 6.5% of patients with MM, with heart failure representing the most frequent manifestation (5.2%). The numerical values of our real-world findings are lower but broadly within the range of previously reported data from large clinical trials, including ASPIRE and ENDEAVOR, which reported higher rates of heart failure (6.4% and 8.2%, respectively) [10,11,12]. These findings are further supported by pooled safety analyses demonstrating a consistent signal of cardiovascular toxicity associated with carfilzomib across randomized and real-world settings [15].

Our multivariate analysis found that pre-existing cardiovascular disease, particularly heart failure and arrhythmias, was associated with an increased risk of carfilzomib-associated CVAEs. Specifically, patients with antecedent heart failure exhibited a 61% increased risk of CVAEs (HR 1.61, 95% CI 1.02–2.56), whereas a history of arrhythmia was associated with a clinically meaningful, but not statistically significant, increase in risk (HR 1.47, 95% CI 0.99–2.20). In contrast, traditional cardiovascular risk factors, including hypertension and dyslipidemia, were not independently associated with CVAEs in this study cohort.

These findings suggest that underlying cardiovascular disease phenotype and reduced functional cardiac reserve may contribute to susceptibility to carfilzomib-associated cardiotoxicity beyond traditional atherosclerotic cardiovascular risk factor burden. Patients with pre-existing cardiovascular disease, including heart failure and arrhythmias, may have diminished physiologic reserve and therefore be less tolerant to the hemodynamic and endothelial stress induced by carfilzomib, predisposing them to clinically significant cardiovascular adverse events. In contrast, traditional cardiovascular risk factors such as hypertension and dyslipidemia may be less predictive of cardiotoxicity in this context. Proteasome inhibition has been linked to endothelial dysfunction, oxidative stress, and impaired myocardial protein homeostasis, providing a biological rationale for these observations [16,17].

Although cardiovascular toxicity associated with carfilzomib has been well described in clinical trials and selected retrospective cohorts, this study adds several clinically relevant contributions. This analysis reflects a contemporary multicenter real-world cohort of patients with multiple myeloma who are older and more comorbid than those enrolled in clinical trials, enhancing external validity. We further demonstrate that pre-existing cardiac disease may be more strongly associated with CVAEs than traditional cardiovascular risk factors, supporting a phenotype-based approach to risk stratification. By focusing on clinically significant events requiring diagnostic evaluation or intervention, our findings emphasize toxicity patterns most relevant to clinical decision-making and likely underestimate lower-grade or subclinical events captured in prospective surveillance studies. Finally, the observed heterogeneity and partial recovery of left ventricular dysfunction provide additional insight into the clinical course and reversibility of carfilzomib-associated cardiotoxicity.

Our results are consistent with prior retrospective studies and real-world datasets, demonstrating that structural and electrical cardiac abnormalities outperform traditional cardiovascular comorbidities in predicting CVAE risk [18,19]. Contemporary cardio-oncology frameworks increasingly emphasize baseline cardiac phenotype and functional reserve over traditional cardiovascular risk factors in predicting treatment-related toxicity [20,21]. Notably, prior prospective studies (e.g., Cornell et al [17].) have demonstrated higher rates of subclinical cardiotoxicity, with CVAEs occurring in 51% of patients receiving carfilzomib-based therapy compared with 17% in those receiving bortezomib, suggesting that the incidence reported here may underestimate the true burden due to reliance on clinically overt events [17].

The timing of carfilzomib-associated CVAEs in our cohort was highly variable, with onset ranging from 3 to 1323 days (median, 157 days), underscoring the heterogeneous and often unpredictable nature of these adverse events. Several factors may explain the relatively prolonged median time to onset observed in our cohort. First, cardiovascular toxicity associated with proteasome inhibition likely develops through multiple mechanisms, including acute endothelial dysfunction and cumulative myocardial injury, resulting in considerable heterogeneity in timing. Second, because our study captured clinically overt events rather than protocol-driven surveillance abnormalities, subclinical cardiac dysfunction may have preceded clinical presentation by weeks to months. Finally, longer treatment duration in some patients allowed delayed toxicities to become clinically apparent. This broad temporal distribution highlights that CVAEs are not confined to early treatment exposure and may occur at any time during therapy. Accordingly, current guidelines recommend longitudinal cardiovascular surveillance throughout the entire course of treatment, particularly for agents associated with delayed or cumulative toxicities [21].

Heart failure events were clinically significant and frequently marked by substantial systolic dysfunction, with a median absolute decline in LVEF of 35%. This degree of reduction reflects a meaningful loss of cardiac reserve and reinforces the potential severity of the cardiac injury associated with carfilzomib.

Encouragingly, partial recovery of cardiac function was observed in a proportion of patients following discontinuation of carfilzomib, with a median LVEF improvement of 20% (range, 0–40). However, recovery was heterogeneous, and a subset of patients experienced minimal or no improvement, suggesting that cardiotoxicity may not be fully reversible. Previous studies have demonstrated the variable reversibility of proteasome inhibitor-associated cardiomyopathy [22,23].

Taken together, these results emphasize the clinical significance and variable reversibility of carfilzomib-associated cardiotoxicity. They further highlight the importance of early detection, prompt intervention, and ongoing cardiac monitoring to optimize outcomes and maximize the likelihood of functional recovery.

Cardiac biomarkers were frequently elevated at the time of cardiovascular adverse events among patients with available data, with marked increases in NT-proBNP and BNP, consistent with significant hemodynamic stress and clinically meaningful heart failure. These findings align with prior studies demonstrating that natriuretic peptides and cardiac troponins are associated with cancer therapy-related cardiac dysfunction and may reflect early myocardial injury in prospective surveillance strategies, although in our cohort biomarker assessment was not systematically performed prior to clinical presentation [19,24].

In this context, the incorporation of cardiac biomarkers into routine monitoring strategies may enhance the identification of subclinical cardiac dysfunction prior to overt clinical deterioration. However, given the retrospective nature of our data and the variability in testing practices, these findings should be interpreted with caution. Prospective studies evaluating the role of serial biomarker measurements as an early warning strategy are, therefore, warranted.

Our findings support the importance of comprehensive cardiovascular assessment before and during carfilzomib therapy. Baseline evaluation should include cardiac history, examination, electrocardiography, echocardiography, and biomarker (troponin, BNP, or NT-proBNP) levels. Multimodal risk assessment incorporating imaging, biomarkers, and clinical factors is recommended by the international cardio-oncology guidelines [20,25]. Our findings further support a risk-adapted approach to the use of carfilzomib. Patients with pre-existing heart failure or impaired cardiac function should undergo careful cardiovascular evaluation before treatment initiation, including assessment of left ventricular ejection fraction and cardiac biomarkers (BNP or NT-proBNP), when available. Although our retrospective study was not designed to establish biomarker or LVEF thresholds for treatment selection, current cardio-oncology guidelines recognize elevated natriuretic peptides and reduced LVEF as markers of increased cardiovascular risk that warrant closer monitoring. Rather than representing absolute contraindications to therapy, these findings should prompt optimization of cardiovascular comorbidities, multidisciplinary discussion with cardio-oncology when appropriate, and consideration of more intensive surveillance throughout treatment. The observed partial recovery in LVEF following discontinuation of carfilzomib in a subset of patients further suggests that treatment-related cardiac dysfunction may be at least partially reversible, emphasizing the importance of early recognition, prompt cardiovascular management, and close follow-up to optimize cardiac recovery. These findings support a pragmatic, risk-adapted approach in which patients with pre-existing heart failure undergo baseline cardiovascular optimization, closer longitudinal monitoring, and, when appropriate, consideration of alternative regimens or dose modifications to reduce cardiotoxicity risk.

CVAEs were associated with numerically inferior overall survival; however, this relationship is likely multifactorial and may reflect underlying patient frailty, treatment interruption, and disease biology rather than a direct causal effect. Because patients with pre-existing cardiovascular disease may have reduced overall survival independent of treatment-related cardiotoxicity, and because the observed difference in survival was not statistically significant, these findings should be interpreted cautiously. Although causality cannot be established, this disparity likely reflects a combination of cardiac dysfunction and treatment interruption or discontinuation. Treatment interruptions due to toxicity are associated with inferior disease control in patients with RRMM [26]. This underscores the importance of proactive cardiovascular management in maintaining treatment continuity.

Close collaboration across oncology, cardiology, and pharmacy disciplines is critical to mitigating CVAEs, preserving treatment continuity, and optimizing both cardiac and oncologic outcomes. Multidisciplinary cardio-oncology care models have been associated with improved outcomes in patients receiving potentially cardiotoxic therapies [27]. In this context, our findings support a pragmatic, risk-adapted approach in which patients with pre-existing heart failure undergo baseline cardiovascular optimization, closer longitudinal monitoring, and, when appropriate, consideration of alternative regimens or dose modifications to reduce cardiotoxicity risk.

This study has several limitations. Its retrospective design introduces potential selection bias and limits causal inference. The relatively small number of CVAEs limited statistical power to identify additional predictors of cardiotoxicity. The limited number of CVAEs also precluded meaningful subgroup analyses evaluating prognosis according to baseline cardiovascular risk status. Detailed information regarding carfilzomib dosing schedules (e.g., once-weekly versus twice-weekly administration), dose modifications, and the rationale for dose adjustments was not consistently available across participating institutions and therefore could not be evaluated as potential contributors to cardiovascular toxicity. In addition, CVAEs were identified based on clinician documentation and clinically overt events rather than standardized toxicity grading systems, which likely resulted in capture of predominantly higher-grade events and under-ascertainment of lower-grade toxicities. Accordingly, the observed incidence is likely lower than that reported in prospective studies that systematically capture both mild and subclinical events. For example, common toxicities such as uncomplicated hypertension were not captured in this analysis, and only more severe manifestations (e.g., hypertensive emergencies) were included. Similarly, echocardiographic and biomarker assessments were more frequently obtained in patients with clinically significant events, introducing ascertainment bias toward more severe cardiotoxicity. The inability to systematically separate the contribution of carfilzomib from other concurrent therapies, including dexamethasone and multi-agent regimens, further limits causal attribution. The relatively small number of CVAEs limited statistical power and constrained multivariable adjustment, increasing the risk of model overfitting and limiting the precision of effect estimates; therefore, hazard ratio estimates should be interpreted cautiously. Biomarker and imaging assessments were not uniformly available across sites, further limiting comprehensive characterization of cardiac dysfunction. Finally, residual confounding—particularly related to treatment selection, disease burden, and patient comorbidities—cannot be excluded.

## 5. Conclusions

Carfilzomib-associated CVAEs, while relatively infrequent, are clinically meaningful and were observed more commonly among patients with pre-existing cardiac dysfunction than among those with traditional cardiovascular risk factors alone. These findings suggest that a phenotype-based cardiovascular risk stratification approach may be useful and reinforce the importance of longitudinal, multidisciplinary cardio-oncology care to optimize outcomes in patients with multiple myeloma receiving carfilzomib.

## Figures and Tables

**Table 1:** Baseline characteristics.

	No CVAEs (*n* = 360)	CVAES (*n* = 25)	*p*-Value
Median age, years (IQR; range)	66 (13; 31–88)	70 (11; 52–83)	0.096
Gender, *n* (%)			
Male	206 (57)	14 (56)	
Race, *n* (%)			0.027
White	282 (78)	17 (68)	
Other	78 (22)	8 (32)	
Median BMI, kg/m^2^ (IQR; range)	28.7 (7.8; 16–48.7)	25.8 (8.3; 18.8–35.7)	0.162
Cardiovascular Diseases, *n* (%)			
Heart failure	38 (11)	9 (36)	0.001
NSTEMI, STEMI, UA *	14 (4)	1 (4)	1.000
Arrhythmias **	51 (14)	14 (56)	0.001
Hypertension	199 (55)	18 (72)	0.103
Diabetes mellitus	70 (19)	8 (32)	0.131
TIA/stroke	3 (1)	1 (4)	0.236
Dyslipidemia	151 (42)	11 (44)	0.840
Tobacco use	141 (39)	9 (36)	0.754
Chronic renal failure	104 (29)	9 (36)	0.450
Median baseline LVEF, % (IQR; range)	60 (9; 49–76)	60 (4; 40–70)	0.605
Type of Multiple Myeloma, *n* (%)			0.046
IgG kappa or lambda	232 (64)	21 (84)	
Non-IgG	128 (36)	4 (16)	

* NSTEMI = non-ST-Segment Elevation Myocardial Infarction; STEMI = ST-elevation Myocardial Infarction; UA = Unstable Angina. ** Arrhythmias included atrial fibrillation, atrial tachycardia, paroxysmal supraventricular tachycardia, premature ventricular contractions, non-sustained ventricular tachycardia, heart block, and other clinically documented rhythm disturbances.

**Table 2:** Previous therapies.

	No CVAEs (*n* = 360)	CVAEs (*n* = 25)	*p*-Value
Number of previous therapies, *n* (%)			0.845
One	94 (26)	7 (28)	
Two	114 (32)	6 (24)	
Three	80 (22)	7 (28)	
Four or more	72 (20)	5 (20)	
Type of previous therapies, *n* (%)			
Anthracyclines	20 (6)	1 (4)	1.000
Alkylating agents	220 (61)	15 (60)	0.912
Lymphodepleting chemotherapy	22 (6)	1 (4)	1.000
Immunomodulating agents	325 (90)	20 (80)	0.103
Proteasome inhibitors	320 (89)	20 (80)	0.181
Mo CD38 Ab	190 (53)	14 (56)	0.755

**Table 3:** Cardiovascular events.

	CVAEs (*n* = 25)
Median time to CVAE, days (IQR; range)	157 (372; 3–1323)
Median cumulative dose carfilzomib, mg (IQR; range)	1366 (3071; 34–6124)
CVAE type, *n* (%)	
Heart failure	20 (86)
Arrhythmias	8 (32)
Pulmonary edema	5 (20)
ACS-NSTEMI *	2 (8)
Death, *n* (%)	
Yes	12 (48)
Cause of Death, *n* (%)	
Progression	9 (75)
Other	3 (25)
Median decline in LVEF at CVAE, % (IQR; range)	35 (31; 20–70)
Median improvement in LVEF after discontinuation, % (IQR; range)	20 (38; 0–40)
	Patients with CVAEs (*n* = 16)
Median BNP at CVAE, pg/mL (IQR; range)	1259 (2742; 88–5771)
	Patients with CVAEs (*n* = 5)
Median NT-proBNP at CVAE, pg/mL (IQR; range)	9329 (15,733; 412–17,500)

* ACS-NSTEMI = Acute Coronary Syndrome Non-ST Elevation Myocardial Infarction.

**Table 4:** Univariable and Multivariable Cox Proportional Hazards Analysis of Baseline Predictors of CVAEs *.

	Patients with Baseline Variable (*n* = 385)	Univariable HR (95% CI)	*p*-Value	Multivariable HR (95% CI)	*p*-Value
Heart failure	47	1.782 (1.165–2.725)	0.008	1.614 (1.016–2.562)	0.042
Hypertension	217	1.123 (0.805–1.566)	0.496	1.072 (0.756–1.520)	0.694
Hyperlipidemia	162	0.944 (0.678–1.314)	0.732	0.841 (0.594–1.190)	0.328
Arrhythmias	65	1.643 (1.127–2.396)	0.010	1.472 (0.992–2.197)	0.055

HR = hazard ratio; CI = confidence interval; number of CVAEs: *n* = 25. * Hazard ratios are derived from the final multivariable Cox proportional hazards model evaluating time from carfilzomib initiation to first cardiovascular adverse event. Covariates were selected a priori based on clinical relevance and cardiovascular risk.

## Data Availability

The data presented in this study are available on request from the corresponding author and with permission from the participating institutions, in accordance with institutional policies and applicable regulations due to institutional privacy and ethical restrictions.
